# High-Intensity Exercise and Geometric Indices of Hip Bone Strength in Postmenopausal Women on or off Bone Medication: The MEDEX-OP Randomised Controlled Trial

**DOI:** 10.1007/s00223-022-00991-z

**Published:** 2022-06-12

**Authors:** Melanie Kistler-Fischbacher, Jedidah S. Yong, Benjamin K. Weeks, Belinda R. Beck

**Affiliations:** 1 Menzies Health Institute Queensland, Griffith University, Gold Coast Campus, QLD 4222 Australia; 2 Exercise Science, School of Health Sciences and Social Work, Griffith University, Gold Coast Campus, QLD 4222 Australia; 3 The Bone Clinic, 26 Turbo Dr, Brisbane, QLD 4151 Australia

**Keywords:** Antiresorptive medication, Bone structure, Exercise, Hip fracture, Osteoporosis, Postmenopausal women

## Abstract

To compare the effects of high-intensity resistance and impact training (HiRIT) to low-intensity, Pilates-based exercise (LiPBE) on proximal femur geometry and explore the influence of antiresorptive medication on those effects. Postmenopausal women with low bone mass, on or off antiresorptive bone medications were randomly allocated, stratified on medication intake, to eight months of twice-weekly, supervised HiRIT (Onero™) or LiPBE (Buff Bones®). 3D hip software was used to analyse proximal femur DXA scans. Outcomes included femoral neck (FN) and total hip (TH), volumetric (e.g. vBMC, vBMD) and geometric (e.g. cortical thickness, cross-sectional area [CSA], section modulus [Z]) indices of bone strength. Data were analysed using analysis of variance. Scans of 102 women were examined: LiPBE, 43; HiRIT, 37; LiPBE-med, 11; HiRIT-med, 11. HiRIT improved TH trabecular vBMC and vBMD (3.1 ± 1.1% versus − 1.2 ± 1.2%, *p* = 0.008; and 1.5 ± 1.0% versus − 1.6 ± 1.2%, *p* = 0.042, respectively) and FN and TH total vBMC (2.0 ± 0.8% versus − 0.2 ± 0.7%, *p* = 0.032; and 0.7 ± 0.4% versus − 0.8 ± 0.6%, *p* = 0.032, respectively), compared to losses in LiPBE. HiRIT also increased Z while LiPBE did not (*p* = 0.035). The combination of HiRIT and medication achieved greater improvements in FN total and trabecular vBMD, total BMC, CSA and Z than HiRIT alone. HiRIT improved geometric parameters of proximal femur strength, while LiPBE exercise was largely ineffective. Medication may enhance some HiRIT effects. Findings suggest reduced hip fracture risk in response to HiRIT.

*Trial registration number* ACTRN12617001511325.

**Supplementary Information:**

The online version contains supplementary material available at 10.1007/s00223-022-00991-z.

## Introduction

Dual-energy X-ray absorptiometry (DXA) to determine areal bone mineral density (aBMD) is standard clinical practice to diagnose osteoporosis [1]. A decrease of 1 SD in the DXA-derived aBMD T-score has been associated with a 50% increase in fracture risk [2]. Nevertheless, approximately, 80% of all fragility fractures occur in individuals with osteopenia or healthy bone mass determined from DXA [3, 4], highlighting the insensitivity of aBMD T-scores for fracture prediction. In fact, while aBMD provides merely an estimate of bone mass, geometric determinants of whole bone strength, such as cortical thickness and cross-sectional area, are directly related to the resistance of a bone to fracture [5].

Three-dimensional parameters of bone strength are commonly examined in research settings using peripheral quantitative computed tomography (pQCT) at the radius and tibia; however, skeletal sites susceptible to the most debilitating osteoporotic fractures, such as the proximal femur (‘hip’) [6], cannot be measured by pQCT. Instead, 3D hip software (DMS Group, Mauguio, France) has been developed to determine trabecular, and cortical bone geometry from proximal femur DXA scans based on 3D modelling that has been validated against QCT images [7]. These DXA-derived parameters of bone geometry and strength can enhance our understanding of the effects of exercise and medications on bone strength beyond aBMD [7]. In fact, deterioration of cortical and trabecular architecture increases fracture risk disproportionally more than aBMD changes detected by DXA [8].

Few exercise trials ( ~10%) in postmenopausal women have included measures of bone geometry and of those findings have been inconclusive. Variability in exercise protocols (e.g. intensity, frequency and duration) and assessment technologies likely contribute to the heterogeneous outcomes [9]. In fact, the positive relationship between load magnitude (exercise intensity) and bone response documented in animal and human studies for aBMD likely also exists for morphological outcomes [10–12]. A direct comparison of the effects of high- and low-intensity exercise on the geometry of a clinically relevant bone site (proximal femur) had not been conducted.

In contrast to the limited and inconclusive data available for exercise trials, the positive effects of antiresorptive bone medications (i.e. bisphosphonates and denosumab) on bone morphology at various skeletal sites have been well documented [13–17]. Findings of a recent meta-analysis suggest that combining exercise and antiresorptive medication may improve proximal femur aBMD more than medication alone [10]; however, data for geometric outcomes are scarce [9].

We recently reported primary and secondary outcomes from the Medication and Exercise for Osteoporosis (MEDEX-OP) randomised controlled trial, including DXA-derived aBMD, anthropometrics, body composition, physical function, adverse events and fall and fracture data [11]. The present work reports secondary outcomes of proximal femur geometry from the same trial. The aim was to determine the effects of high-intensity resistance and impact training (HiRIT) with or without antiresorptive bone medication, compared to a low-intensity, Pilates-based exercise programme (LiPBE) with or without antiresorptive bone medication, on proximal femur bone geometry in postmenopausal women with low bone mass. We hypothesised that HiRIT would improve parameters of bone geometry and strength, whereas LiPBE would not. We further hypothesised that bone-targeted exercise combined with antiresorptive medication would yield greater benefits than exercise alone.

## Methods

### Study Design

The MEDEX-OP trial was an eight-month, partially blinded, randomised controlled trial, conducted between March 2018 and August 2020. Postmenopausal women who were on or off stable doses of antiresorptive bone medication therapy were randomly allocated to HiRIT or LiPBE, stratified by medication intake, resulting in four groups (HiRIT, LiPBE, HiRIT-med, LiPBE-med). The trial was approved by the Griffith University Human Research Ethics Committee (approval no.: 2017/739) and prospectively registered on the Australian New Zealand Clinical Trial Registry (ACTRN12617001511325). All study procedures were conducted in accordance with the NHMRC National Statement on Ethical Conduct in Human Research and the Declaration of Helsinki, and written informed consent was obtained from all participants. No animal studies were performed in the course of these experiments. The full study protocol has been published [18], and DXA-derived aBMD, functional performance, anthropometric and body composition outcomes as well as incident falls, fractures and adverse events have been reported [11].

### Study Participants and Allocation

We recruited healthy women who were at least five years post menopause, had low bone mass (lumbar spine [LS] and/or femoral neck [FN] *T*-score of ≤ − 1.0) and were on or off stable doses of bisphosphonate (i.e. alendronate, risedronate or zoledronic acid) or denosumab therapy for at least 12 months prior to enrolment. The following exclusion criteria applied: current or previous (< 12 months prior to enrolment) therapy with anabolic bone medication (e.g. teriparatide), hormone therapy, or selective estrogen receptor modulators; other medications or medical conditions known to influence bone health (e.g. glucocorticoids, diabetes); recent fracture, injury or medical condition that could prevent completion of the exercise programme; regular strength, resistance or high-impact training (≥ 1/wk); lifestyle interventions that could interfere with the study (e.g. weight loss); inability or unwillingness to attend twice-weekly exercise classes or planned absence of more than three weeks during the study intervention.

Eligible participants were block randomised (block size of four), stratified by presence or absence of bone medication intake, with a 1:1 allocation ratio to HiRIT or LiPBE. A computer-generated randomisation sequence was created, and sequentially numbered, sealed opaque envelopes were prepared by an independent person prior to study commencement. Allocation was concealed from the tester and participant until completion of initial testing. Participants could not be blinded to the exercise intervention; however, they were blinded to the study hypotheses (i.e. which exercise programme was expected to be most beneficial).

The current report includes observations from the 102 of the original 115 participants of the MEDEX-OP trial [11] who were scanned on a Medix DXA machine (Medix DR, Medilink, France) for which the 3D hip software was available. The first 13 participants were scanned on a Norland DXA (Norland XR-800, Norland Medical Systems, Inc., Trumbull, CT, USA) that does not perform 3D hip analyses and data could therefore not be included in the current analysis.

### Exercise Interventions

A detailed description of the exercise training protocols has been published elsewhere [11, 18]. Both exercise protocols included twice-weekly, 40-min sessions on non-consecutive days for eight months (35 weeks). All sessions took place at The Bone Clinic, Brisbane, Australia and were supervised by a qualified Exercise Scientist or Physiotherapist. Compliance with the exercise programme was recorded and calculated as a percentage of the maximum 70 sessions attended.

#### High-Intensity Resistance and Impact Training (HiRIT, Onero™)

The Onero™ programme includes three resistance, one impact and two balance exercises each session. The resistance training exercises (deadlift, back squat, overhead press) were conducted in 5 sets of 5 repetitions at > 80% 1RM. Training intensity was monitored using a 6 to 20 Borg scale to achieve a rating of ≥ 16 for each exercise, corresponding to ‘very hard’. The impact exercise involved an assisted jump and a stiff-legged landing with minimal shock attenuation on landing. All four exercises were gradually introduced during a two-week accommodation period with a focus on technique with no or minimal loads.

#### Low-Intensity, Pilates-Based Exercise (LiPBE, Buff Bones®)

The Buff Bones® movement system focuses on whole body strengthening, mobility and balance. The majority of the programme includes Pilates-based exercises performed on the mat in supine, prone, side-lying and quadruped position. The last 10–15 min of each session were performed in a standing weight bearing position and consisted of body weight squats, balance and low-impact exercises (i.e. heel drops and stomping) as well as exercises performed with light dumbbells (e.g. bicep curl, tricep extension, bent-over row). Six to ten repetitions were performed for each exercise.

### Anthropometrics and Lifestyle Characteristics

Height was measured barefoot with a wall-mounted stadiometer (Seca 216, Seca, Hamburg, Germany) and weight was obtained using a digital scale (Charder MS 3200, Charder, Taichung City, Taiwan). Body mass index (BMI) was calculated per the accepted formula. Calcium intake from food, beverages and supplements was assessed using the AusCal diet questionnaire, and average daily intake was estimated using an online calculator (https://calciumcalculator.com.au/) [19]. The Bone-specific Physical Activity Questionnaire (BPAQ) was used to assess past, current and total physical activity of relevance to bone health [20].

### Dual-Energy X-ray Absorptiometry

Proximal femur scans of the skeletally non-dominant leg were acquired using standard DXA positioning and protocol (Medix DR, Medilink, France) at baseline and eight months. The machine was calibrated daily and all scans were performed by a single DXA technician. Analyses were performed by the same, unblinded investigator, but verified by two independent investigators who were off site and blinded to group allocation. The scans were analysed using 3D Hip software (DMS Group, Mauguio, France), according to manufacturer guidelines. To run the 3D analysis, three markers were placed at anatomical landmarks on a standard 2D image; at the distal edge of the lesser trochanter, and at the superior and inferior junctions of the neck and head of the femur. The marker positions are used by the software to compare the 2D scans to existing QCT reference scans to produce a participant-specific shape and density model of the proximal femur and to estimate structural and geometric parameters of bone strength [7]. All FN and total hip (TH) outcomes produced by the 3D Hip software were derived and analysed: FN and TH trabecular, cortical and total bone mineral content (BMC), volume, and volumetric bone mineral density (vBMD); FN medial, lateral and total cortical thickness and TH total cortical thickness; FN cross-sectional area (CSA), cross-sectional moment of inertia (CSMI) and section modulus (Z). The coefficients of variation (CVs) for short-term measurement reliability for 3D BMC, vBMD and volume outcomes in a sample of women aged 55+ years range from 0.02 to 0.94% at the TH and 0.20 to 1.77% for FN in our laboratory. The range was 0.45 to 4.55% for cortical thickness and 0.14 to 3.2% for cross-sectional outcomes.

### Statistical Analyses

Differences in descriptive participant characteristics at baseline were examined using one-way ANOVA for normally distributed data, Kruskal–Wallis test for non-normally distributed continuous data and chi-square tests for categorical data. Because of the small sample size in the medication groups, main effect analyses comparing the two exercise interventions, irrespective of medication intake, were conducted (i.e. combined HiRIT plus HiRIT-med groups versus LiPBE plus LiPBE-med groups). There were no baseline differences in any characteristic between the two groups. Unadjusted, repeated measures analysis of variance (RMANOVA) and one-way analysis of variance (ANOVA) were therefore used to compare between-group differences in eight-month absolute and percentage change, respectively. Comparison of baseline characteristics between the four groups (parsed on medication use) yielded significant differences in age (Table 1). Exploratory subgroup analyses were therefore adjusted for baseline age, using analysis of covariance (RMANCOVA and one-way ANCOVA). P values were reported for all subgroup comparisons; however, only comparisons related to our study hypotheses were reported in detail, namely: LiPBE versus HiRIT, LiPBE-med versus HiRIT-med, HiRIT-med versus HiRIT and LiPBE-med versus LiPBE.

**Table 1:** Participant characteristics at baseline

Characteristic	LiPBE (*n* = 43)	HiRIT (*n* = 37)	LiPBE-med (*n* = 11)	HiRIT-med (*n* = 11)	*p* value
Age, years	63.7 ± 4.9	63.8 ± 6.1	65.3 ± 7.5	70.6 ± 5.6^a,b,c^	0.006
Weight, kg	67.4 ± 11.2	69.5 ± 13.1	64.7 ± 13.5	61.1 ± 4.8	0.231
Height, cm	162.7 ± 5.1	162.1 ± 5.1	160.3 ± 7.5	157.8 ± 6.3	0.053
BMI, kg/cm^2^	25.6 ± 4.6	26.4 ± 4.8	25.1 ± 4.8	24.6 ± 2.1	0.597
Osteoporosis medications					
Bisphosphonates, *n* (%)			3 (27%)	2 (18%)	
Denosumab, *n* (%)			8 (73%)	9 (82%)	
Femoral neck aBMD g/cm^2^	0.712 ± 0.064	0.719 ± 0.110	0.694 ± 0.075	0.686 ± 0.075	0.449
Femoral neck, *T*-score	− 1.8 ± 0.5	− 1.8 ± 0.9	− 2.0 ± 0.6	− 2.0 ± 0.6	0.458
Total hip aBMD, g/cm^2^	0.841 ± 0.084	0.826 ± 0.107	0.800 ± 0.076	0.820 ± 0.078	0.540
Total hip, *T*-score	− 1.3 ± 0.6	− 1.4 ± 0.7	− 1.6 ± 0.5	− 1.5 ± 0.5	0.543
Total BPAQ score, unitless	23.7 ± 22.2	17.6 ± 14.5	16.9 ± 10.4	20.5 ± 20.7	0.663
Calcium intake, mg/day	1045 ± 492	891 ± 372	1230 ± 528	1065 ± 392	0.132

Data are mean ± SD

*aBMD* areal bone mineral density, *HiRIT* high-intensity resistance and impact training, *HiRIT-med* high-intensity resistance and impact training plus bone medications, *LiPBE* low-intensity Pilates-based exercise, *LiPBE-med* low-intensity Pilates-based exercise plus bone medications

^a^*p* ≤ 0.05 compared to LiPBE

^b^*p* ≤ 0.05 compared to HiRIT

^c^*p* ≤ 0.05 compared to LiPBE-med

Per protocol (PP) and intention-to-treat (ITT) analyses were undertaken for main effects and subgroup analyses; however, due to space limitations and the exploratory nature of the subgroup analyses, only ITT results are reported for the latter. Participants who completed the eight-month trial with exercise compliance ≥ 70% were included in PP analyses. For ITT analyses, data of all participants were included and missing values were imputed based on the mean percentage change of the respective group. The Bonferroni method was applied to all analyses to adjust for multiple comparisons.

Reported in the tables of this manuscript are *p* values from ANOVA or ANCOVA. P values from RMANOVA/RMANCOVA are not reported due to space limitations, but mirror those from one-way ANOVA or ANCOVA, unless stated otherwise. Results for baseline characteristics are presented as mean ± SD, whereas all results from ANOVA and ANCOVA are presented as mean ± SE. Statistical analyses were undertaken using SPSS software (version 26.0; IBM Inc., Chicago, IL, USA) with significance level set at *p* ≤ 0.05. The investigator who entered the data and performed statistical analyses was not blinded to treatment allocation.

## Results

### Participants

DXA-derived 3D hip analysis outcomes were available for 102 participants; LiPBE *n* = 43, HiRIT *n* = 37, LiPBE-med *n* = 11, HiRIT-med *n* = 11. Of those, 93 completed the eight-month exercise intervention (nine were lost to follow up). Five LiPBE participants withdrew due to a study-related adverse event (*n* = 2), family commitments (*n* = 1), unrelated medical condition (*n* = 1) and loss of interest (*n* = 1). One HiRIT participant withdrew due to a study-related adverse event and three HiRIT-med participants withdrew due to unwillingness to attend the exercise classes due to the COVID-19 pandemic (*n* = 1), unrelated medical condition (*n* = 1) and loss of interest (*n* = 1). Baseline characteristics of participants who withdrew from the trial did not differ from those who completed the eight-month intervention. Adverse events, falls and fracture data have been reported in detail previously [11].

At baseline, average age of the combined sample was 64.7 ± 6.0 years and average femoral neck T-score was − 1.8 ± 0.7. Participant characteristics for each study arm at baseline are presented in Table 1. Mean age in the HiRIT-med group was slightly higher than the other three groups. To account for the difference, all subgroup analyses were adjusted for baseline age. There were no differences in baseline characteristics between the combined HiRIT and LiPBE groups so main effect analyses were unadjusted.

Exercise compliance was similar for all groups (LiPBE 81.9 ± 13.3%, HiRIT 83.3 ± 10.3%, LiPBE-med 79.8 ± 16.2%, HiRIT-med 86.3 ± 12.1%, *p* = 0.690).

### Main Effect Analyses

Eight-month change in volumetric BMC (vBMC), volume and volumetric BMD (vBMD) outcomes from main effects analyses (ITT) is presented in Table 2. Results from one-way ANOVA of percent change revealed HiRIT increased total FN vBMC but LiPBE did not (2.0 ± 0.8% versus − 0.2 ± 0.7%, *p* = 0.032). Similarly, HiRIT improved TH trabecular vBMC and vBMD and total vBMC, compared to losses in the LiPBE group (3.1 ± 1.1% versus − 1.2 ± 1.2%, *p* = 0.008; 1.5 ± 1.0% versus − 1.6 ± 1.2%, *p* = 0.042; and 0.7 ± 0.4% versus − 0.8 ± 0.6%, *p* = 0.032, respectively). Examination of within-group change similarly showed the HiRIT group improved FN total vBMC (0.059 ± 0.023 g, *p* = 0.011), TH trabecular vBMC (0.216 ± 0.084 g, *p* = 0.011), FN and TH trabecular volume (0.162 ± 0.062 cm^3^, *p* = 0.011; and 0.859 ± 0.367 cm^3^, *p* = 0.022, respectively), FN cortical volume (0.054 ± 0.022 cm^3^, *p* = 0.016) and FN and TH total volume (0.216 ± 0.068 cm^3^, *p* = 0.002; and 1.011 ± 0.401 cm^3^, *p* = 0.014, respectively), but lost FN and TH cortical vBMD (− 8.8 ± 3.3 g/cm^3^ and -12.1 ± 3.2 g/cm^3^, *p* < 0.001, respectively). LiPBE lost FN and TH cortical vBMD (− 10.3 ± 3.1 g/cm^3^, *p* = 0.001 and − 10.0 ± 3.0 g/cm^3^, *p* = 0.001, respectively) and total vBMD (− 3.9 ± 1.8 g/cm^3^, *p* = 0.037 and − 3.6 ± 1.4 g/cm^3^, *p* = 0.010, respectively), but gained FN trabecular volume (0.117 ± 0.057 cm^3^, *p* = 0.045) and FN total volume (0.129 ± 0.063 cm^3^, *p* = 0.044).

**Table 2:** Baseline and eight-month measures with percent change in volumetric outcomes at the femoral neck and total hip from main effect analyses (ITT analysis, *n* = 102)

	LiPBE (*n* = 54)			HiRIT (*n* = 48)			
Outcome measure	Baseline	Follow up	% change (95% CI)	Baseline	Follow up	% change (95% CI)	*p* value
Trabecular							
FN vBMC, g	1.614 ± 0.048	1.609 ± 0.047	− 0.0 ± 1.1 (− 2.2, 2.1)	1.598 ± 0.050	1.634 ± 0.050	3.3 ± 1.6 (0.0, 6.6)	0.083
FN volume, cm^3^	10.110 ± 0.261	10.227 ± 0.260	1.3 ± 0.7 (− 0.1, 2.6)	10.351 ± 0.282	10.513 ± 0.280	1.4 ± 0.7 (0.1, 2.8)	0.882
FN vBMD, g/cm^3^	161.0 ± 4.6	159.0 ± 4.5	− 1.2 ± 1.1 (− 3.4, 1.1)	160.2 ± 4.9	161.3 ± 4.7	2.0 ± 1.6 (− 1.2, 5.2)	0.104
TH vBMC, g	7.337 ± 0.199	7.239 ± 0.213	− 1.2 ± 1.2 (− 3.6, 1.1)	7.189 ± 0.211	7.404 ± 0.226 *	3.1 ± 1.1 (0.9, 5.2)	**0.008**
TH volume, cm^3^	58.882 ± 1.438	59.058 ± 1.441	0.7 ± 0.5 (− 0.4, 1.8)	61.003 ± 1.550	61.862 ± 1.554	1.4 ± 0.6 (0.1, 2.7)	0.411
TH vBMD, g/cm^3^	129.0 ± 3.1	126.8 ± 3.2	− 1.6 ± 1.2 (− 3.9, 0.7)	125.0 ± 3.3	126.6 ± 3.9	1.5 ± 1.0 (− 0.4, 3.5)	**0.042**
Cortical							
FN vBMC, g	1.738 ± 0.035	1.731 ± 0.035	− 0.2 ± 0.7 (− 1.6, 1.2)	1.738 ± 0.037	1.762 ± 0.038	1.4 ± 0.7 (− 0.1, 2.9)	0.122
FN volume, cm^3^	2.543 ± 0.052	2.555 ± 0.054	1.3 ± 0.7 (− 0.2, 2.8)	2.589 ± 0.056	2.643 ± 0.058 *	2.8 ± 0.9 (0.9, 4.6)	0.210
FN vBMD, g/cm^3^	686.3 ± 7.8	676.0 ± 7.5 *	− 1.4 ± 0.4 (− 2.2, 0.6)	687.3 ± 8.3	678.5 ± 7.9 *	− 1.2 ± 0.5 (− 2.2, − 0.2)	0.780
TH vBMC, g	11.094 ± 0.222	11.030 ± 0.214	− 0.4 ± 0.5 (− 1.4, 0.6)	11.036 ± 0.235	10.946 ± 0.227	− 0.8 ± 0.5 (− 1.7, 0.2)	0.602
TH volume, cm^3^	16.133 ± 0.304	16.301 ± 0.300	1.2 ± 0.5 (0.2, 2.2)	16.243 ± 0.328	16.395 ± 0.323	1.2 ± 0.6 (0.0, 2.4)	0.941
TH vBMD, g/cm^3^	739.2 ± 7.7	729.2 ± 7.4 *	− 1.3 ± 0.4 (− 2.0, − 0.5)	738.9 ± 8.2	726.8 ± 7.9 *	− 1.6 ± 0.5 (− 2.5, − 0.6)	0.614
Total							
FN vBMC, g	3.352 ± 0.073	3.340 ± 0.073	− 0.2 ± 0.7 (0.7, − 1.6)	3.336 ± 0.077	3.395 ± 0.077 *	2.0 ± 0.8 (0.4, 3.5)	**0.032**
FN volume, cm^3^	12.653 ± 0.300	12.782 ± 0.298 *	1.2 ± 0.6 (0.1, 2.3)	12.940 ± 0.323	13.156 ± 0.322 *	1.7 ± 0.6 (0.4, 3.0)	0.547
FN vBMD, g/cm^3^	267.3 ± 5.6	263.5 ± 5.5 *	− 1.3 ± 0.7 (− 2.7, 1.1)	266.2 ± 6.0	266.3 ± 5.8	0.4 ± 0.5 (− 1.6, 0.5)	0.100
TH vBMC, g	18.431 ± 0.391	18.268 ± 0.394	− 0.8 ± 0.6 (− 2.0, 0.3)	18.225 ± 0.415	18.350 ± 0.417	0.7 ± 0.4 (− 0.1, 1.6)	**0.032**
TH volume, cm^3^	75.015 ± 1.697	75.356 ± 1.686	0.8 ± 0.4 (− 0.1, 1.6)	77.246 ± 1.829	78.257 ± 1.818 *	1.4 ± 0.6 (0.2, 2.5)	0.399
TH vBMD, g/cm^3^	261.0 ± 4.6	257.4 ± 4.4 *	− 1.3 ± 0.6 (− 2.5, − 0.1)	255.7 ± 4.8	254.3 ± 4.7	0.4 ± 0.5 (− 1.4, 0.5)	0.265

Data are mean ± SE

*P* values represent between-group comparison of % change from one-way ANOVA

*CI* confidence interval, *FN* femoral neck, *HiRIT* high-intensity resistance and impact training, *ITT* intention-to-treat, *LiPBE* low-intensity Pilates-based exercise, *TH* total hip, *vBMC* volumetric bone mineral content, *vBMD* volumetric bone mineral density

^*^within-group change from baseline *p* ≤ 0.05 from RMANOVA

Results for geometric and cross-sectional outcomes from ITT analyses are presented in Table 3. There were no between-group differences from one-way ANOVA; however, results from RMANOVA revealed HiRIT increased Z compared to a loss in LiPBE (0.011 ± 0.005 cm^3^ versus − 0.004 ± 0.005 cm^3^, *p* = 0.035). Within-group effects indicated that HiRIT increased FN total and medial cortical thickness (0.028 ± 0.010 mm, *p* = 0.009 and 0.058 ± 0.020 mm, *p* = 0.040, respectively), CSMI (0.021 ± 0.009 cm^4^, *p* = 0.020), and Z (0.011 ± 0.005 cm^3^, *p* = 0.035). LiPBE increased FN medial cortical thickness (0.040 ± 0.019 mm, *p* = 0.037), and TH total thickness (0.015 ± 0.007 mm, *p* = 0.040) but no other index of bone strength.

**Table 3:** Baseline and eight-month measures with percent change in geometric and cross-sectional outcomes at the femoral neck and total hip from main effect analyses (ITT analysis, *n* = 102)

	LiPBE (*n* = 54)			HiRIT (*n* = 48)			
Outcome measure	Baseline	Follow up	% change (95% CI)	Baseline	Follow up	% change (95% CI)	*p* value
Cortical thickness							
FN total, mm	1.464 ± 0.016	1.477 ± 0.018	1.0 ± 0.7 (− 0.4, 2.5)	1.460 ± 0.017	1.487 ± 0.019 *	1.8 ± 0.7 (0.4, 3.2)	0.426
FN medial, mm	2.424 ± 0.030	2.465 ± 0.034 *	1.8 ± 0.8 (0.3, 3.3)	2.436 ± 0.032	2.495 ± 0.036 *	2.4 ± 0.9 (0.6, 4.1)	0.625
FN lateral, mm	0.959 ± 0.013	0.957 ± 0.016	− 0.0 ± 1.1 (− 2.2, 2.2)	0.963 ± 0.014	0.980 ± 0.017	1.7 ± 0.8 (0.1, 3.4)	0.213
TH total, mm	1.745 ± 0.014	1.760 ± 0.015 *	0.9 ± 0.4 (0.0, 1.8)	1.726 ± 0.015	1.731 ± 0.016	0.3 ± 0.4 (− 0.5, 1.1)	0.339
Cross-sectional outcomes							
FN CSA, cm^2^	0.754 ± 0.015	0.753 ± 0.015	0.1 ± 0.8 (− 1.5, 1.7)	0.751 ± 0.016	0.762 ± 0.016	1.6 ± 0.8 (0.1, 3.2)	0.171
FN CSMI, cm^4^	0.964 ± 0.027	0.968 ± 0.027	0.8 ± 1.0 (− 1.2, 2.8)	0.959 ± 0.029	0.981 ± 0.028 *	2.3 ± 0.9 (0.6, 4.1)	0.265
FN Z, cm^3^	0.526 ± 0.012	0.522 ± 0.012	− 0.3 ± 1.0 (− 2.4, 1.7)	0.522 ± 0.013	0.533 ± 0.013 *	2.2 ± 0.9 (0.4, 3.9)	0.071

Data are mean ± SE

*P* values represent between-group comparison of % change from one-way ANOVA

^*^within-group change from baseline *p* ≤ 0.05 from RMANOVA

*CI* confidence interval, *CSA* cross-sectional area, *CSMI* cross-sectional moment of inertia, *FN* femoral neck, *HiRIT* high-intensity resistance and impact training, *ITT* intention-to-treat, *LiPBE* low-intensity Pilates-based exercise, *TH* total hip, *Z* section modulus

Results from PP main effects analyses are presented in Supplementary Table 1 for volumetric outcomes and Supplementary Table 2 for geometric and cross-sectional outcomes. The between-group differences for FN total vBMC and TH trabecular vBMD were no longer significant but otherwise mirror ITT results.

### Subgroup Analyses

**Fig. 1:**
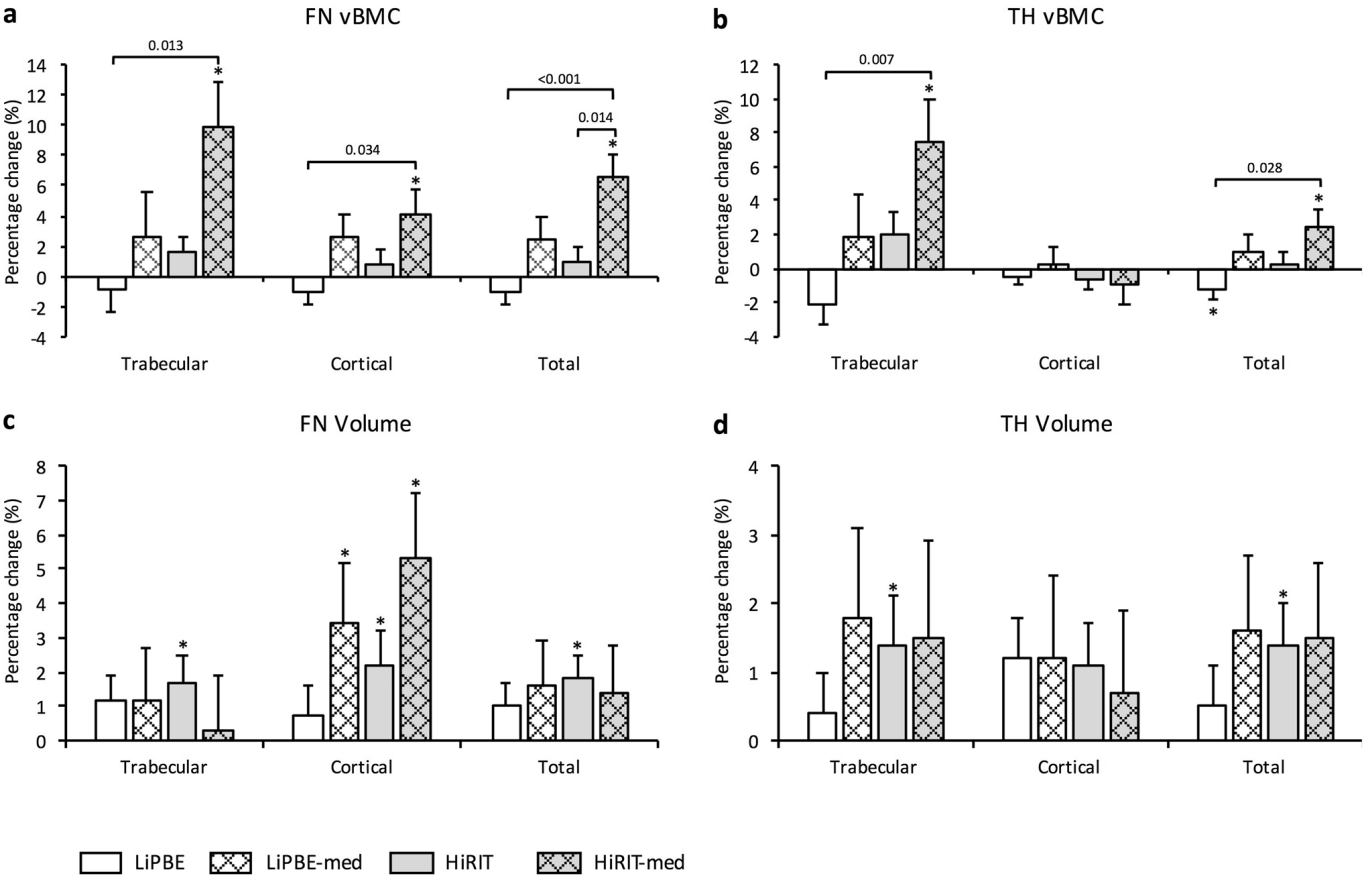
Eight-month percent change (mean ± SE) from exploratory subgroup analyses in femoral neck and total hip vBMC (**a**) and (**b**) and volume (**c**) and (**d**). ITT data; LiPBE *n* = 43, LiPBE-med *n* = 11, HiRIT = 37, HiRIT-med *n* = 11. * Indicates within-group change from baseline from RMANCOVA (*p* ≤ 0.05); *FN* femoral neck, *HiRIT* high-intensity resistance and impact training, *HiRIT*-med high-intensity resistance and impact training plus bone medications, *ITT* intention-to-treat, *LiPBE* low-intensity Pilates-based exercise, *LiPBE-med* low-intensity Pilates-based exercise plus bone medications, *TH* total hip, *vBMC* volumetric bone mineral content

**Fig. 2:**
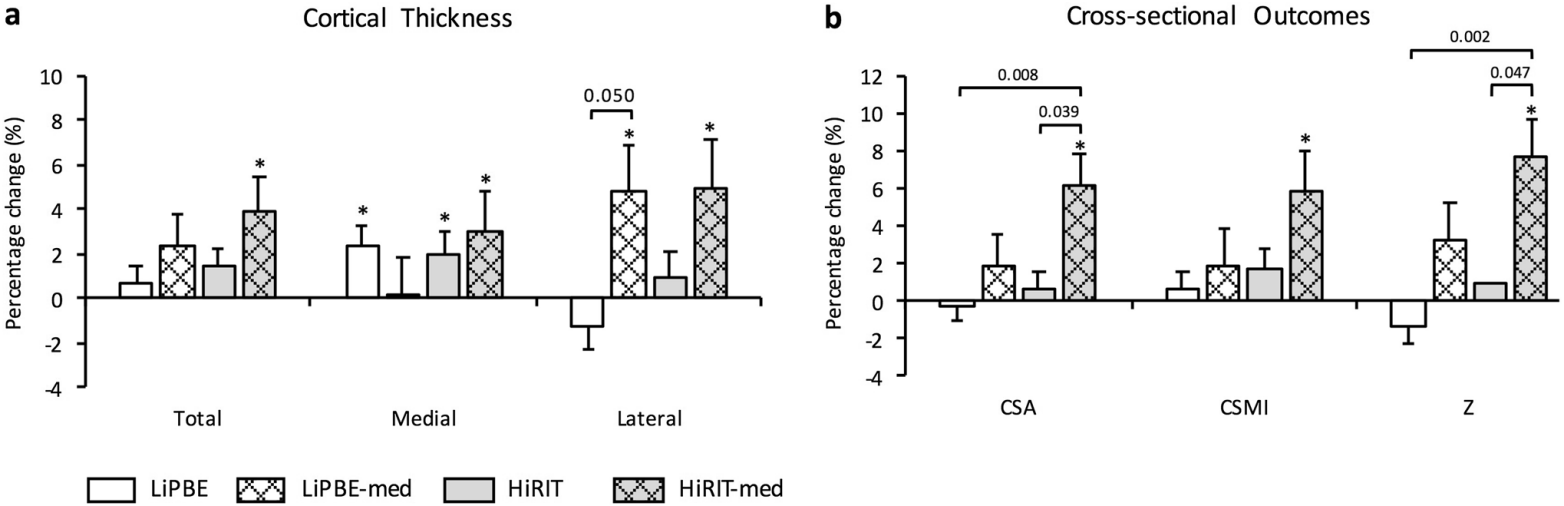
Eight-month percent change (mean ± SE) from exploratory subgroup analyses in femoral neck geometric (**a**) and cross-sectional (**b**) outcomes. ITT data; LiPBE *n* = 43, LiPBE-med *n* = 11, HiRIT = 37, HiRIT-med *n* = 11. * Indicates within-group change from baseline from RMANCOVA (*p* ≤ 0.05); *CSA* cross-sectional area, *CSMI* cross-sectional moment of inertia, *HiRIT* high-intensity resistance and impact training, *HiRIT-med* high-intensity resistance and impact training plus bone medications, *ITT* intention-to-treat, *LiPBE* low-intensity Pilates-based exercise, *LiPBE-med* low-intensity Pilates-based exercise plus bone medications, *Z* section modulus

Results from subgroup analyses (ITT) are presented graphically in Figs. 1 and 2. Complete results are presented in Supplementary Table 3 for volumetric outcomes and Supplementary Table 4 for geometric and cross-sectional outcomes.

#### No Medication Subgroup Outcomes

There were no between-group differences for HiRIT and LiPBE groups not on medications for any of the volumetric outcomes (Fig. 1). Within-group analysis revealed HiRIT gained FN and TH trabecular volume (0.154 ± 0.070 cm^3^, *p* = 0.029; and 0.820 ± 0.389 cm^3^, *p* = 0.038, respectively), FN cortical volume (0.055 ± 0.022 cm^3^, *p* = 0.016), FN and TH total volume (0.979 ± 0.481 cm^3^, *p* = 0.021; and 0.210 ± 0.075 cm^3^, *p* = 0.007, respectively), but lost FN and TH cortical vBMD (− 8.7 ± 3.8 g/cm^3^, *p* = 0.024; and − 11.0 ± 3.7 g/cm^3^, *p* = 0.004, respectively). LiPBE lost TH trabecular vBMD (− 2.932 ± 1.452 g/cm^3^, *p* = 0.046), FN and TH cortical vBMD (− 11.978 ± 3.519 g/cm^3^, *p* = 0.001, and 11.088 ± 3.465 g/cm^3^, *p* = 0.002, respectively), FN and TH total vBMD (− 5.694 ± 1.046 g/cm^3^, *p* = 0.004, and − 4.145 ± 1.538 g/cm^3^, *p* = 0.008, respectively) and TH total vBMC (− 0.242 ± 0.098 g, *p* = 0.016).

Similarly, there were no between-group differences for geometric and cross-sectional outcomes (Fig. 2), but HiRIT improved FN medial cortical thickness (0.051 ± 0.023 mm, *p* = 0.028), while LiPBE improved FN medial cortical thickness (0.055 ± 0.021 mm, *p* = 0.012) and TH total cortical thickness (0.017 ± 0.008 mm, *p* = 0.032).

#### Medication Subgroup Outcomes

There were no between-group differences for the HiRIT-med versus LiPBE-med subgroups. However, HiRIT-med improved volumetric outcomes more than HiRIT for FN trabecular vBMD (9.5 ± 3.0% versus 0.0 ± 1.5, *p* = 0.040), and FN total vBMC and vBMD (6.5 ± 1.6% versus 0.9 ± 0.8%, *p* = 0.014; and 5.0 ± 1.5% versus − 0.8 ± 0.8%, *p* = 0.008, respectively; Fig. 1). There were no significant differences between LiPBE-med and LiPBE for volumetric outcomes (Fig. 1). Within-group analyses revealed HiRIT-med increased FN and TH trabecular vBMC (0.107 ± 0.040 g, *p* = 0.009; and 0.513 ± 0.183 g, *p* = 0.006, respectively), FN and TH trabecular vBMD (11.113 ± 3.995 g/cm^3^, *p* = 0.007; and 0.298 ± 3.025 g/cm^3^, *p* = 0.040, respectively), FN cortical vBMC (0.067 ± 0.028 g, *p* = 0.018), FN cortical volume (0.114 ± 0.044 cm^3^, *p* = 0.010), TH cortical vBMD (− 16.567 ± 7.217 g/cm^3^, *p* = 0.024) and FN and TH total vBMC (0.174 ± 0.048 g, *p* = 0.000; and 0.419 ± 0.205 g, *p* = 0.043, respectively). LiPBE-med increased FN cortical volume (0.085 ± 0.041 cm^3^, *p* = 0.042).

With respect to geometric and strength outcomes, HiRIT-med improved FN CSA (6.1 ± 1.7% versus 0.6 ± 0.9%, *p* = 0.039) and Z (7.6 ± 2.1% versus 1.0 ± 1.1%, *p* = 0.047) more than HiRIT alone (Fig. 2). LiPBE-med increased lateral cortical thickness more than LiPBE (4.8 ± 2.0% versus − 1.3 ± 1.0%, *p* = 0.050; Fig. 2). Within-group observations indicated HiRIT-med increased FN total, medial and lateral cortical thickness (0.055 ± 0.023 mm, *p* = 0.019; 0.090 ± 0.044 mm, *p* = 0.046; and 0.046 ± 0.022 mm, *p* = 0.036, respectively), FN CSA (0.039 ± 0.012 cm^2^, *p* = 0.002), FN CSMI (0.047 ± 0.020 cm^4^, p = 0.023) and FN Z (0.034 ± 0.011 cm^3^, *p* = 0.002), while LiPBE-med increased FN lateral cortical thickness (0.048 ± 0.020 mm, *p* = 0.019).

## Discussion

The MEDEX-OP trial compared the efficacy of a high-intensity resistance and impact exercise programme (HiRIT, Onero™) with a low-intensity, Pilates-based exercise programme (LiPBE, Buff Bones®) on parameters of bone geometry at the proximal femur in postmenopausal women with low bone mass or osteoporosis. We also explored the influence of bone medication on the efficacy of each exercise programme. Based on main effect analyses, combining individuals on and off medications, HiRIT increased a number of indices of proximal femur bone geometry and strength, whereas LiPBE had little effect. HiRIT combined with antiresorptive medication increased some indices of bone strength more than HiRIT alone. Both programmes were well accepted with good compliance (HiRIT: 83%; LiPBE: 82%) and were largely safe (adverse events: HiRIT, 4; LiPBE, 3) [11].

The positive effects of HiRIT on trabecular and total vBMC and vBMD from 3D hip analysis complement the trend for a superior effect of HiRIT over LiPBE on FN and TH aBMD from standard DXA reported elsewhere [11]. While an abundance of evidence exists on the effect of bone-targeted exercise on areal BMC and BMD, few have examined cortical and trabecular compartments separately, or if they did, peripheral measurement sites were used [9]. Only two previous randomised controlled trials have examined bone volume outcomes at the proximal femur in postmenopausal women [21, 22]. The first involved a 12-month, high-impact circuit training programme including drop jumps, skipping and hopping plus some upper body resistance training exercises, and did not improve vBMD at the proximal femur [21]. The second study was the LIFTMOR (Lifting Intervention For Training Muscle and Osteoporosis Rehabilitation) trial, which tested the same HiRIT intervention as the MEDEX-OP trial and reported an increase in FN cortical vBMC [22]. While no significant between-group differences were reported for this outcome in the present trial, we observed a non-significant net benefit of 1.6% in favour of HIRIT versus control (*p* = 0.122), which is comparable to the LIFTMOR results (net benefit of HiRIT versus control of 1.5%, *p* = 0.028) [22].

Although we did not detect significant between-group differences in percent change in FN cortical thickness, cross-sectional area and stiffness index, there was a significant between-group difference in absolute change in section modulus Z (bending strength) in favour of HiRIT (*p* = 0.035), from RMANOVA. This observation was reinforced by positive within-group changes in cortical thickness, CSMI and section modulus in the HiRIT group. Cortical thickness, CSMI and section modulus, important determinants of bone strength, have been associated with femoral neck failure load [23, 24] and fracture risk [25–27]. Women with a recent hip fracture had up to 30% thinner femoral neck cortex than fracture-free women [28]. Our observed improvements in femoral neck geometry following HiRIT are therefore highly clinically relevant. The LIFTMOR and LIFTMOR for men (LIFTMOR-M) trials similarly reported 6.3% and 5.7% net benefit in femoral neck cortical thickness following eight months of HiRIT compared with control in postmenopausal women and middle-aged and older men [22, 29]. Thus, although the available evidence is limited, taken together, it suggests that HiRIT increases femoral neck cortical thickness, a highly clinically relevant finding in respect to fracture risk. Although we observed positive within-group changes for FN total volume and cortical thickness at the medial FN and total TH following LiPBE, the lack of positive effect at 18 other sites and losses observed at 4 sites leads us to conclude that LiPBE does not provide a notable stimulus for proximal femur bone geometry.

Similarly, only six randomised controlled trials have reported the effect of exercise on CSA, moment of inertia and Z (section modulus, an index of bending strength) at the proximal femur [21, 30–34], and of those, only two have reported positive effects [30, 31]. One of the trials found a 3.2% increase in Z in the exercise limb, compared to a 0.8% loss in the control limb, following a 6-month unilateral intervention consisting of multi-directional hopping [31]. The second reported a net benefit for section modulus of 5% following 12 months of resistance training (8–10 repetitions at 70–80% 1 RM) compared to another exercise intervention including the same resistance training protocol plus impact exercises (e.g. jumping) [30]. These results are counterintuitive and may be explained by relatively small sample sizes as the authors indicate the effect was not confirmed by an efficacy analysis.

The influence of antiresorptive medication on exercise effects, a clinically relevant outcome of interest, was investigated in exploratory subgroup analyses as the number of participants on medication who volunteered for the study was small. Antiresorptive medications enhanced the effects of HiRIT on multiple outcomes, including trabecular and total vBMC and vBMD, and FN cross-sectional area and section modulus. By contrast, LiPBE plus medication improved only FN lateral cortical thickness compared to LiPBE alone. Although we did not observe statistically significant differences between the HiRIT-med and LiPBE-med groups for any of the outcome measures, HiRIT-med yielded greater effects compared to LiPBE-med and the non-medication groups, particularly for vBMC, vBMD and cross-sectional outcomes. A factorial design with adequate sample sizes would be required to fully test for an interaction and/or additive effects of HiRIT and antiresorptive medication, both of which have been reported to independently improve proximal femur bone structure and geometry [14, 22]. Two trials have previously examined the independent and combined effects of antiresorptive agents and exercise therapy on bone structural outcomes in postmenopausal women and yielded inconclusive results [21, 34]. Both trials used a 2 × 2 factorial design and a 12-months impact protocol. High-impact training combined with hormone therapy (estradiol plus norethisterone acetate) increased cortical vBMD at the tibial shaft more than exercise alone; however, no positive influence of hormone therapy plus exercise compared to exercise alone was reported for any other outcomes (i.e. vBMD and moment of inertia at the proximal and mid femur and tibia) [21]. Similarly, the second trial reported no additive or interactive effect of exercise and alendronate treatment [34]. Neither of the two exercise protocols provoked a remarkable effect applied in isolation, which may have contributed to a lack of interaction effect with antiresorptive agents. Our preliminary results may suggest that a high-intensity exercise stimulus (i.e. HiRIT) is required to observe an interaction effect between exercise and antiresorptive medications.

Low-intensity Pilates-like training operated as something of a control group in the current study, being largely ineffective for bone. We were therefore able to compare, albeit in an exploratory manner, the effect of antiresorptive therapy (i.e. LiPBE-med) with HiRIT alone and observed that in many cases the effects were similar. The greater effect of HiRIT on other indices of fracture risk (e.g. back and leg muscle strength, functional mobility, stature) than ‘control’ (LiPBE) [11] further supports the use of HiRIT as osteoporosis therapy. Furthermore, we observed HiRIT was safe, even for individuals on bone medication, who often have a particularly high risk of fracture. Close ongoing supervision and individual load progression is crucial to safe application of high-intensity exercise in individuals at high fracture risk.

To our knowledge, this is the first study to directly compare the effects of high and low-intensity, bone-targeted exercise on indices of proximal femur bone geometry in postmenopausal women with low bone mass. Furthermore, this trial provides novel data specific to women on antiresorptive osteoporosis therapy, who are frequently excluded from exercise trials [9]. The examination of trabecular and cortical hip geometry and indices of bone strength is another strength of the present trial, since the majority of previous exercise trials in postmenopausal women have reported only areal BMD or measured indices of bone strength at peripheral sites of less clinical relevance [9]. While the 3D hip software does not provide a direct measure of geometry, outcomes have been well validated against QCT images (correlation coefficients for vBMD = 0.80–0.93) so its use is increasingly accepted [14, 35].

Several study limitations warrant acknowledgement. The convenience sampling of women on existing medication therapy rather than allocation to de novo therapy may have introduced some bias. It is possible there are variations in characteristics related to adoption of medication, treatment duration and pharmacological properties of the different types of medications. Also, conclusions from subgroup analyses of medication groups are limited by small group numbers. Our sample included relatively healthy women which may limit generalisability of our findings; however, we believe our participants represent a large proportion of women with osteoporosis at high fracture risk. It also should be noted that, while blinding was applied wherever possible, it was not feasible to fully blind investigators nor to blind participants to exercise group allocation which may have introduced some bias. This was managed by blinding group allocation to investigators until after baseline assessments were completed (i.e. randomisation was performed following baseline data collection), blinding participants to the study hypotheses to limit expectation bias, and by verification of DXA analyses by blinded investigators.

In conclusion, high-intensity resistance and impact training improved indices of proximal femur bone strength, whereas low-intensity, Pilates-based training was largely ineffective. Our findings add to the increasing evidence that high-intensity exercise provokes a greater osteogenic response than lower intensity [9, 10]. Preliminary analyses suggest that medication intake may enhance effects of high-intensity exercise; however, a larger trial including novel exposure to medication is needed to fully examine the effect.

## Supplementary Information

Below is the link to the electronic supplementary material.

Supplementary file1 (PDF 193 KB)

## References

[1] (1994) Assessment of fracture risk and its application to screening for postmenopausal osteoporosis. Report of a WHO Study Group. World Health Organ Tech Rep Ser 843:1-129 7941614

[2] Hillier TA, Stone KL, Bauer DC, Rizzo JH, Pedula KL, Cauley JA (2007). Evaluating the value of repeat bone mineral density measurement and prediction of fractures in older women: the study of osteoporotic fractures. Arch Intern Med.

[3] Chapurlat R, Bui M, Sornay-Rendu E, Zebaze R, Delmas PD, Liew D (2020). Deterioration of cortical and trabecular microstructure identifies women with osteopenia or normal bone mineral density at imminent and long-term risk for fragility fracture: a prospective study. J Bone Miner Res.

[4] Samelson EJ, Broe KE, Xu H, Yang L, Boyd S, Biver E (2019). Cortical and trabecular bone microarchitecture as an independent predictor of incident fracture risk in older women and men in the Bone Microarchitecture International Consortium (BoMIC): a prospective study. Lancet Diabetes Endocrinol.

[5] Ammann P, Rizzoli R (2003). Bone strength and its determinants. Osteoporos Int.

[6] Cummings SR, Melton LJ (2002). Epidemiology and outcomes of osteoporotic fractures. Lancet.

[7] Humbert L, Martelli Y, Fonolla R, Steghofer M, Di Gregorio S, Malouf J (2017). 3D-DXA: assessing the femoral shape, the trabecular macrostructure and the cortex in 3D from DXA images. IEEE Trans Med Imaging.

[8] Sornay-Rendu E, Boutroy S, Duboeuf F, Chapurlat RD (2017). Bone microarchitecture assessed by HR-pQCT as predictor of fracture risk in postmenopausal women: the OFELY study. J Bone Miner Res.

[9] Kistler-Fischbacher M, Weeks BK, Beck BR (2021). The effect of exercise intensity on bone in postmenopausal women (part 1): a systematic review. Bone.

[10] Kistler-Fischbacher M, Weeks BK, Beck BR (2021). The effect of exercise intensity on bone in postmenopausal women (part 2): a meta-analysis. Bone.

[11] Kistler-Fischbacher M, Yong JS, Weeks BK, Beck BR (2021). A Comparison of bone-targeted exercise with and without antiresorptive bone medication to reduce indices of fracture risk in postmenopausal women with low bone mass: the MEDEX-OP randomized controlled trial. J Bone Miner Res.

[12] Rubin CT, Lanyon LE (1985). Regulation of bone mass by mechanical strain magnitude. Calcif Tissue Int.

[13] Seeman E, Delmas PD, Hanley DA, Sellmeyer D, Cheung AM, Shane E (2010). Microarchitectural deterioration of cortical and trabecular bone: differing effects of denosumab and alendronate. J Bone Miner Res.

[14] Winzenrieth R, Humbert L, Di Gregorio S, Bonel E, García M, Del Rio L (2018). Effects of osteoporosis drug treatments on cortical and trabecular bone in the femur using DXA-based 3D modeling. Osteoporos Int.

[15] Burghardt AJ, Kazakia GJ, Sode M, de Papp AE, Link TM, Majumdar S (2010). A longitudinal HR-pQCT study of alendronate treatment in postmenopausal women with low bone density: relations among density, cortical and trabecular microarchitecture, biomechanics, and bone turnover. J Bone Miner Res.

[16] Zebaze R, Libanati C, McClung MR, Zanchetta JR, Kendler DL, Høiseth A (2016). Denosumab reduces cortical porosity of the proximal femoral shaft in postmenopausal women with osteoporosis. J Bone Miner Res.

[17] Recker RR, Delmas PD, Halse J, Reid IR, Boonen S, García-Hernandez PA (2008). Effects of intravenous zoledronic acid once yearly on bone remodeling and bone structure. J Bone Miner Res.

[18] Fischbacher M, Weeks BK, Beck BR (2019). The influence of antiresorptive bone medication on the effect of high-intensity resistance and impact training on osteoporotic fracture risk in postmenopausal women with low bone mass: protocol for the MEDEX-OP randomised controlled trial. BMJ Open.

[19] Beck BR, Weeks BK, Norling TL (2011) A novel Australian calcium-specific diet questionnaire: validity and reliability. Osteoporos Int 22(Suppl. 4):S626.

[20] Weeks BK, Beck BR (2008). The BPAQ: a bone-specific physical activity assessment instrument. Osteoporos Int.

[21] Cheng S, Sipilä S, Taaffe DR, Puolakka J, Suominen H (2002). Change in bone mass distribution induced by hormone replacement therapy and high-impact physical exercise in post-menopausal women. Bone.

[22] Watson SL, Weeks BK, Weis LJ, Harding AT, Horan SA, Beck BR (2018). High-intensity resistance and impact training improves bone mineral density and physical function in postmenopausal women with osteopenia and osteoporosis: the LIFTMOR randomized controlled trial. J Bone Miner Res.

[23] Bousson V, Le Bras A, Roqueplan F, Kang Y, Mitton D, Kolta S (2006). Volumetric quantitative computed tomography of the proximal femur: relationships linking geometric and densitometric variables to bone strength. Role for compact bone Osteoporos Int.

[24] Manske SL, Liu-Ambrose T, de Bakker PM, Liu D, Kontulainen S, Guy P (2006). Femoral neck cortical geometry measured with magnetic resonance imaging is associated with proximal femur strength. Osteoporos Int.

[25] Ahlborg HG, Nguyen ND, Nguyen TV, Center JR, Eisman JA (2005). Contribution of hip strength indices to hip fracture risk in elderly men and women. J Bone Miner Res.

[26] Johannesdottir F, Poole KE, Reeve J, Siggeirsdottir K, Aspelund T, Mogensen B (2011). Distribution of cortical bone in the femoral neck and hip fracture: a prospective case-control analysis of 143 incident hip fractures; the AGES-REYKJAVIK study. Bone.

[27] Glüer CC, Cummings SR, Pressman A, Li J, Glüer K, Faulkner KG (1994). Prediction of hip fractures from pelvic radiographs: the study of osteoporotic fractures. The study of osteoporotic fractures research group. J Bone Miner Res.

[28] Poole KE, Treece GM, Mayhew PM, Vaculík J, Dungl P, Horák M (2012). Cortical thickness mapping to identify focal osteoporosis in patients with hip fracture. PLoS ONE.

[29] Harding AT, Weeks BK, Lambert C, Watson SL, Weis LJ, Beck BR (2020). Effects of supervised high-intensity resistance and impact training or machine-based isometric training on regional bone geometry and strength in middle-aged and older men with low bone mass: The LIFTMOR-M semi-randomised controlled trial. Bone.

[30] Karinkanta S, Heinonen A, Sievänen H, Uusi-Rasi K, Pasanen M, Ojala K (2007). A multi-component exercise regimen to prevent functional decline and bone fragility in home-dwelling elderly women: randomized, controlled trial. Osteoporos Int.

[31] Hartley C, Folland JP, Kerslake R, Brooke-Wavell K (2020). High-impact exercise increased femoral neck bone density with no adverse effects on imaging markers of knee osteoarthritis in postmenopausal women. J Bone Miner Res.

[32] Chilibeck PD, Vatanparast H, Pierson R, Case A, Olatunbosun O, Whiting SJ (2013). Effect of exercise training combined with isoflavone supplementation on bone and lipids in postmenopausal women: a randomized clinical trial. J Bone Miner Res.

[33] Duff WR, Kontulainen SA, Candow DG, Gordon JJ, Mason RS, Taylor-Gjevre R (2016). Effects of low-dose ibuprofen supplementation and resistance training on bone and muscle in postmenopausal women: a randomized controlled trial. Bone reports.

[34] Uusi-Rasi K, Kannus P, Cheng S, Sievanen H, Pasanen M, Heinonen A (2003). Effect of alendronate and exercise on bone and physical performance of postmenopausal women: a randomized controlled trial. Bone.

[35] Winzenrieth R, Ominsky MS, Wang Y, Humbert L, Weiss RJ (2021). Differential effects of abaloparatide and teriparatide on hip cortical volumetric BMD by DXA-based 3D modeling. Osteoporos Int.

