# Supplementary material for: High-Intensity Exercise and Geometric Indices of Hip Bone Strength in Postmenopausal Women on or off Bone Medication: The MEDEX-OP Randomised Controlled Trial
Source: Calcif Tissue Int. 2022 Jun 12;111(3):256–66. doi: 10.1007/s00223-022-00991-z (PMC9188729; doi:10.1007/s00223-022-00991-z)
Supplement: Supplementary file 1 — Supplementary file1 (PDF 193 KB) [file 223_2022_991_MOESM1_ESM.pdf]

SUPPLEMENTARY MATERIAL

Supplementary Table 1. Baseline and eight-month measures with percent change in volumetric outcomes at the femoral neck and total hip (PP analysis, n = 78)

Outcome measure	LiPBE (n = 39)			HiRIT (n = 39)			*p* value
	Baseline	Follow up	% change (95% CI)	Baseline	Follow up	% change (95% CI)	
Trabecular							
FN vBMC, g	1.564 ± 0.056	1.559 ± 0.056	0.0 ± 1.4 (-2.9, 2.9)	1.617 ± 0.056	1.632 ± 0.056	1.4 ± 1.2 (-1.0, 3.8)	0.444
FN volume, cm^3	9.869 ± 0.291	9.975 ± 0.285	1.3 ± 0.9 (-0.6, 3.2)	10.101 ± 0.291	10.196 ± 0.285	1.2 ± 0.8 (-0.4, 2.8)	0.953
FN vBMD, g/cm^3	160.2 ± 5.8	158.5 ± 5.7	-1.1 ± 1.4 (-4.0, 1.8)	164.1 ± 5.8	163.4 ± 5.7	0.3 ± 1.2 (-2.0, 2.6)	0.447
TH vBMC, g	7.149 ± 0.239	6.986 ± 0.259	-2.2 ± 1.5 (-5.2, 0.8)	7.189 ± 0.239	7.350 ± 0.259	2.2 ± 1.2 (-0.2, 4.6)	**0.024**
TH volume, cm^3	58.467 ± 1.613	58.669 ± 1.601	0.5 ± 0.7 (-0.9, 2.0)	60.025 ± 1.613	60.812 ± 1.601 *	1.5 ± 0.7 (-0.0, 3.0)	0.353
TH vBMD, g/cm^3	127.2 ± 3.9	124.2 ± 4.0	-2.2 ± 1.5 (-5.3, 0.9)	126.4 ± 3.9	127.0 ± 4.0	0.7 ± 1.0 (-1.3, 2.7)	0.111
Cortical							
FN vBMC, g	1.693 ± 0.041	1.689 ± 0.041	-0.0 ± 0.8 (-1.7, 1.6)	1.746 ± 0.041	1.770 ± 0.041	1.4 ± 0.9 (-0.4, 3.2)	0.245
FN volume, cm^3	2.496 ± 0.056	2.528 ± 0.058	1.5 ± 0.9 (-0.4, 3.4)	2.544 ± 0.056	2.609 ± 0.058 *	2.7 ± 1.1 (0.5, 4.9)	0.406
FN vBMD, g/cm^3	679.1 ± 9.2	668.9 ± 8.8 *	-1.4 ± 0.6 (-2.5, -0.3)	688.2 ± 9.2	679.8 ± 8.8 *	-1.2 ± 0.6 (-2.4, 0.0)	0.747
TH vBMC, g	10.784 ± 0.259	10.668 ± 0.252	-0.9 ± 0.5 (-2.0, 0.2)	10.999 ± 0.259	10.946 ± 0.252	-0.5 ± 3.4 (0.6, -1.6)	0.607
TH volume, cm^3	15.891 ± 0.331	16.019 ± 0.327	0.9 ±0.6 (-0.3, 2.1)	16.038 ± 0.331	16.239 ± 0.327 *	1.4 ± 0.7 (0.0, 2.8)	0.571
TH vBMD, g/cm^3	730.8 ± 9.1	719.9 ± 8.6 *	-1.4 ± 0.5 (-2.4, -0.4)	738.8 ± 9.1	727.5 ± 8.6 *	-1.5 ± 0.6 (-2.6, -0.3)	0.941
Total							
FN vBMC, g	3.257 ± 0.085	3.248 ± 0.087	-0.1 ± 0.9 (-1.8, 1.6)	3.362 ± 0.085	3.402 ± 0.087	1.2 ± 0.6 (-0.1, 2.4)	0.224
FN volume, cm^3	12.365 ± 0.334	12.503 ± 0.328	1.2 ± 0.7 (-0.3, 2.8)	12.645 ± 0.334	12.805 ± 0.328 *	1.5 ± 0.7 (0.0, 3.1)	0.778
FN vBMD, g/cm^3	266.1 ± 7.0	262.5 ± 6.9	-1.2 ± 0.9 (-3.1, 0.6)	270.7 ± 7.0	269.6 ± 6.9	-0.3 ± 0.6 (-1.6, 1.0)	0.380
TH vBMC, g	17.933 ± 0.460	17.654 ± 0.466	-1.5 ± 0.7 (-2.8, -0.1)	18.188 ± 0.460	18.296 ± 0.466	0.6 ± 0.5 (-0.3, 1.5)	**0.012**
TH volume, cm^3	74.358 ± 1.892	74.688 ±1.869	0.5 ± 0.5 (-0.5, 1.6)	76.064 ± 1.892	77.051 ± 1.869 *	1.5 ± 0.7 (0.1, 2.8)	0.286
TH vBMD, g/cm^3	257.4 ± 5.6	252.9 ± 5.4 *	-1.7 ± 0.8 (-3.2, -0.1)	257.3 ± 5.6	255.4 ± 5.4	-0.7 ± 0.5 (-1.7, 0.4)	0.278

Abbreviations: CI, confidence interval; FN, femoral neck; HiRIT, high-intensity resistance and impact training; ITT, intention-to-treat; LiPBE, low-intensity Pilates-based exercise; TH, total hip; vBMC, volumetric bone mineral content; vBMD, volumetric bone mineral density
Data are mean ± SE
p values represent between-group comparison of % change from one-way ANOVA
* Within-group change from baseline $p \leq 0.05$ from RMANOVA

Supplementary Table 2. Baseline and eight-month measures with percent change in geometric and cross-sectional outcomes at the femoral neck and total hip (PP analysis, n = 78)

Outcome measure	LiPBE (n = 54) Baseline	LiPBE (n = 54) Follow up	LiPBE (n = 54) % change (95% CI)	HiRIT (n = 48) Baseline	HiRIT (n = 48) Follow up	HiRIT (n = 48) % change (95% CI)	*p* value
Cortical thickness							
FN total, mm	1.462 ± 0.020	1.479 ± 0.022	1.3 ± 0.9 (-0.6, 3.2)	1.473 ± 0.020	1.502 ± 0.022 *	1.9 ± 0.8 (0.2, 3.6)	0.660
FN medial, mm	2.411 ± 0.037	2.462 ± 0.042 *	2.3 ± 1.0 (0.3, 4.3)	2.455 ± 0.037	2.511 ± 0.042 *	2.2 ± 1.0 (0.2, 4.3)	0.959
FN lateral, mm	0.968 ± 0.016	0.968 ± 0.019	0.2 ± 1.4 (-2.7. 3.1)	0.974 ± 0.016	0.996 ± 0.019	2.0 ± 1.0 (0.1, 4.0)	0.295
TH total, mm	1.731 ± 0.018	1.743 ± 0.019	0.8 ± 0.6 (-0.4, 1.9)	1.729 ± 0.018	1.736 ± 0.019	0.4 ± 0.5 (-0.5, 1.3)	0.603
Cross-sectional outcomes							
FN CSA, cm^2	0.737 ± 0.018	0.738 ± 0.019	0.4 ± 1.0 (-1.7, 2.4)	0.762 ± 0.018	0.768 ± 0.019	0.9 ± 0.7 (-0.5, 2.2)	0.681
FN CSMI, cm^4	0.927 ± 0.033	0.934 ± 0.032	1.3 ± 1.3 (-1.3, 3.9)	0.970 ± 0.033	0.985 ± 0.032	1.8 ± 1.0 (-0.1, 3.7)	0.748
FN Z, cm^3	0.513 ± 0.015	0.511 ± 0.015	0.1 ± 1.3 (-2.6, 2.7)	0.531 ± 0.015	0.538 ± 0.015	1.3 ± 0.9 (-0.4, 3.0)	0.443

Abbreviations: CI, confidence interval; CSA, cross-sectional area; CSMI, cross-sectional moment of inertia; FN, femoral neck; ITT, intention-to-treat; LiPBE, low-intensity Pilates-based exercise; TH, total hip; Z, section modulus
Data are mean ± SE
P values represent between-group comparison of % change from one-way ANOVA
* Within-group change from baseline $p \leq 0.05$ from RMANOVA

Supplementary Table 3. Baseline and eight-month measures with percent change in volumetric outcomes at the femoral neck and total hip from exploratory subgroup analyses (ITT analysis, n = 102)

	LiPBE (n = 43)			HiRIT (n = 37)			LiPBE-med (n = 11)			HiRIT-med (n = 11)			*p* values						
Outcome measure	Baseline	Follow up	% change	Baseline	Follow up	% change	Baseline	Follow up	% change	Baseline	Follow up	% change	Omnibus	HiRIT vs. LiPBE	HiRIT-med vs. LiPBE-med	HiIRT-med vs. HiRIT	LiPBE-med vs. LiPBE	HiRIT-med vs. LiPBE	LiPBE-med vs. HiRIT
**Trabcecular**																			
FN vBMC, g	1.622 ± 0.054	1.607 ± 0.053	-0.9 ± 1.5 (-3.8, 2.0)	1.609 ± 0.058	1.626 ± 0.057	1.6 ± 1.6 (-1.5, 4.8)	1.556 ± 0.105	1.586 ± 0.104	2.6 ± 2.9 (-3.1, 8.3)	1.589 ± 0.112	1.696 ± 0.110 *	9.9 ± 3.0 (3.8, 15.9)	**0.022**	1.000	0.510	0.117	1.000	**0.013**	1.000
FN volume, cm^3	10.103 ± 0.271	10.213 ± 0.269	1.2 ± 0.7 (-0.3, 2.7)	10.345 ± 0.292	10.499 ± 0.290 *	1.7 ± 0.8 (0.1 ± 3.3)	10.157 ± 0.533	10.247 ± 0.530	1.2 ± 1.5 (-1.7, 4.1)	9.651 ± 0.565	9.671 ± 0.561	0.3 ± 1.6 (-2.7, 3.4)	0.883	1.000	1.000	1.000	1.000	1.000	1.000
FN vBMD, g/cm^3	161.6 ± 5.2	158.3 ± 5.0	-2.0 ± 1.4 (-4.8, 0.8)	158.7 ± 5.6	157.2 ± 5.4	0.0 ± 1.5 (-3.0, 3.1)	156.4 ± 10.3	159.5 ± 9.8	1.9 ± 2.8 (-3.7, 7.4)	167.5 ± 10.9	178.6 ± 10.4 *	9.5 ± 3.0 (3.6, 15.4)	**0.010**	1.000	0.390	**0.040**	1.000	**0.005**	1.000
TH vBMC, g	7.356 ± 0.225	7.199 ± 0.241	-2.1 ± 1.2 (-4.5, 0.3)	7.135 ± 0.242	7.282 ± 0.259	2.0 ± 1.3 (-0.6, 4.6)	7.164 ± 0.443	7.285 ± 0.473	1.9 ± 2.4 (-2.8, 6.7)	7.472 ± 0.469	7.985 ± 0.501 *	7.5 ± 2.5 (2.4, 12.5)	**0.006**	0.134	0.676	0.380	0.837	**0.007**	1.000
TH volume, cm^3	58.842 ± 1.523	58.977 ± 1.520	0.4 ± 0.6 (-0.9, 1.6)	60.967 ± 1.639	61.787 ± 1.537 *	1.4 ± 0.7 (0.1, 2.8)	60.109 ± 2.995	61.037 ± 2.991	1.8 ± 1.3 (-0.7, 4.3)	58.777 ± 3.171	29.523 3.166	1.5 ± 1.4 (-1.2, 4.2)	0.596	1.000	1.000	1.000	1.000	1.000	1.000
TH vBMD, g/cm^3	129.3 ± 3.5	126.3 ± 3.6 *	-2.1 ± 1.2 (-4.4, 0.2)	123.2 ± 3.8	123.6 ± 3.9	0.6 ± 1.3 (-1.9, 3.1)	126.1 v 6.9	126.9 ± 7.0	0.6 ± 2.3 (-3.9, 5.2)	132.9 ± 7.3	139.2 ± 7.5 *	5.5 ± 2.3 (0.7, 10.3)	**0.041**	0.700	0.869	0.460	1.000	**0.037**	1.000
**Cortical**																			
FN vBMC, g	1.763 ± 0.039	1.742 ± 0.040	-1.0 ± 0.8 (-2.5, 0.6)	1.776 ± 0.042	1.791 ± 0.043	0.8 ± 0.8 (-0.8, 2.5)	1.646 ± 0.077	1.688 ± 0.078	2.6 ± 1.5 (-0.4, 5.6)	1.605 ± 0.082	1.672 ± 0.083 *	4.1 ± 1.6 (0.9, 7.3)	**0.019**	0.701	1.000	0.435	0.234	**0.034**	1.000
FN volume, cm^3	2.544 ± 0.052	2.558 ± 0.054	0.7 ± 0.9 (-1.1, 2.5)	2.590 ± 0.056	2.645 ± 0.058 *	2.2 ± 1.0 (0.3, 4.1)	2.505 ± 0.102	2.589 ± 0.105 *	3.4 ± 1.8 (-0.1, 6.9)	2.339 ± 0.108	2.453 ± 0.112 *	5.3 ± 1.9 (1.6, 9.1)	0.139	1.000	1.000	0.865	1.000	0.188	1.000
FN vBMD, g/cm^3	692.4 ± 8.8	680.4 ± 8.5 *	-1.6 ± 0.5 (-2.6, -0.6)	686.8 ± 9.5	678.2 ± 9.1 *	-1.2 ± 0.6 (-2.3, -0.1)	662.1 ±17.4	657.8 ±16.7	-0.6 ± 1.0 (-2.6, 1.3)	689.1 ± 18.4	681.7 ± 17.7	-1.0 ± 1.1 (-3.1 ± 1.1)	0.821	1.000	1.000	1.000	1.000	1.000	1.000
TH vBMC, g	11.208 ± 0.249	11.126 ± 0.242	-0.5 ± 0.5 (-1.6, 0.5)	11.146 ± 0.268	11.055 ± 0.261	-0.7 ± 0.6 (-1.9, 0.4)	10.568 ± 0.490	10.601 ± 0.476	0.3 ± 1.0 (-1.8, 2.3)	10.745 ± 0.519	10.642 ± 0.504	-1.0 ± 1.1 (-3.2, 1.2)	0.835	1.000	1.000	1.000	1.000	1.000	1.000
TH volume, cm^3	16.107 ± 0.313	16.282 ± 0.308	1.2 ± 0.6 (0.0, 2.4)	16.218 ± 0.337	16.377 ± 0.332	1.1 ± 0.6 (-0.2, 2.4)	16.177 ± 0.616	16.389 ± 0.607	1.2 ± 1.2 (-1.1, 3.6)	15.688 ± 0.652	15.906 ± 0.642	1.7 ± 1.2 (-0.8, 4.1)	0.984	1.000	1.000	1.000	1.000	1.000	1.000
TH vBMD, g/cm^3	745.2 ± 8.7	734.1 ± 8.4*	-1.4 ± 0.5 (-2.3, -0.5)	738.7 ± 9.4	727.7 ± 9.0*	-1.5 ± 0.5 (-2.5, -0.5)	714.0 ± 17.2	708.5 ± 16.5	-0.8 ± 0.9 (-2.6, 1.1)	740.9 ± 18.2	724.3 ± 17.4 *	-2.1 ± 1.0 (-4.1, -0.2)	0.773	1.000	1.000	1.000	1.000	1.000	1.000

Supplementary Table 3 continued.

	LiPBE (n = 43)			HiRIT (n = 37)			LiPBE-med (n = 11)			HiRIT-med (n = 11)			*p* values						
Outcome measure	Baseline	Follow up	% change	Baseline	Follow up	% change	Baseline	Follow up	% change	Baseline	Follow up	% change	Omnibus	HiRIT vs. LiPBE	HiRIT-med vs. LiPBE-med	HiRIT-med vs. LiPBE-med	LiPBE-med vs. LiPBE	HiRIT-med vs. LiPBE	LiPBE-med vs. HiRIT
**Total**																			
FN vBMC, g	3.385 ± 0.082	3.349 ± 0.082	-1.0 ± 0.8 (-2.5, 0.5)	3.385 ± 0.088	3.417 ± 0.089	0.9 ± 0.8 (-0.7, 2.5)	3.203 ± 0.161	3.273 ± 0.162	2.5 ± 1.5 (-0.5, 5.4)	3.195 ± 0.171	3.369 ± 0.172 *	6.5 ± 1.6 (3.4, 9.6)	**< 0.001**	0.488	0.378	**0.014**	0.230	**< 0.001**	0.230
FN volume, cm^3	12.647 ± 0.310	12.770 ± 0.309	1.0 ± 0.7 (-0.3, 2.3)	12.935 ± 0.334	13.145 ± 0.332 *	1.8 ± 0.7 (0.4, 2.3)	12.662 ± 0.611	12.837 ± 0.607	1.6 ± 1.3 (-1.0, 4.1)	11.990 ± 0.647	12.131 ± 0.643	1.4 ± 1.4 (-1.3, 4.1)	0.875	1.000	1.000	1.000	1.000	1.000	1.000
FN vBMD, g/cm^3	269.3 ± 6.4	263.6 ± 6.2 *	-2.0 ± 0.7 (-3.5, -0.5)	265.4 ± 6.9	262.8 ± 6.6	-0.8 ± 0.8 (-2.4, 0.8)	258.2 ± 12.6	261.3 ± 12.1	1.2 ± 1.5 (-1.7, 4.0)	270.2 ± 13.4	281.8 ± 12.9 *	5.0 ± 1.5 (1.9, 8.0)	**0.001**	1.000	0.431	**0.008**	0.340	0.001	1.000
TH vBMC, g	18.564 ± 0.442	18.322 ± 0.447 *	-1.3 ± 0.5 (-2.3, -0.2)	18.281 ± 0.476	18.337 ± 0.481	0.3 ± 0.6 (-0.8, 1.5)	17.733 ± 0.869	17.886 ± 0.879	0.9 ± 1.1 (-1.2, 3.0)	18.218 ± 0.920	18.637 ± 0.930 *	2.4 ± 1.1 (0.2, 4.7)	**0.017**	0.305	1.000	0.627	0.455	**0.028**	1.000
TH volume, cm^3	74.949 ± 1.790	75.256 ± 1.778	0.5 ± 0.6 (-0.6, 1.6)	77.185 ± 1.927	78.164 ± 1.914 *	1.4 ± 0.6 (0.2, 2.5)	76.286 ± 3.521	77.425 ± 3.497	1.6 ± 1.1 (-0.6, 3.7)	74.466 ± 3.728	75.437 ± 3.703	1.5 ± 1.1 (-0.7, 3.8)	0.646	1.000	1.000	1.000	1.000	1.000	1.000
TH vBMD, g/cm^3	262.7 ± 5.2	258.5 ± 5.0 *	-1.5 ± 0.6 (-2.7, -0.3)	254.2 ± 5.5	252.0 ± 5.4	-0.8 ± 0.6 (-2.1, 0.5)	252.5 ± 10.1	251.6 ± 9.9	-0.3 ± 1.2 (-2.6, 2.0)	262.8 ± 10.7	264.5 ± 10.4	0.9 ± 1.2 (-1.6, 3.3)	0.354	1.000	1.000	1.000	1.000	0.525	1.000

Abbreviations: CI, confidence interval; FN, femoral neck; HiRIT, high-intensity resistance and impact training; HiRIT-med, high-intensity resistance and impact training plus medications; ITT, intention-to-treat; LiPBE, low-intensity Pilates-based exercise; LiPBE-med, low-intensity Pilates-based exercise plus medications; TH, total hip; vBMC, volumetric bone mineral content; vBMD, volumetric bone mineral density

Data are mean ± SE

p values represent between-group comparison of % change from one-way ANCOVA, adjusted for age at baseline

* Within-group change from baseline $p \leq 0.05$ from RMANCOVA

Supplementary Table 4. Baseline and eight-month measures with percent change in geometric and cross-sectional outcomes at the femoral neck and total hip from exploratory subgroup analyses (ITT analysis, n = 102)

	LiPBE (n = 43)			HiRIT (n = 37)			LiPBE-med (n = 11)			HiRIT-med (n = 11)			*p* values						
	Baseline	Follow up	% change	Baseline	Follow up	% change	Baseline	Follow up	% change	Baseline	Follow up	% change	Omnibus	HiRIT vs. LiPBE	HiRIT-med vs. LiPBE-med	HiRIT-med vs. HiRIT	LiPBE-med vs. LiPBE	HiRIT-med vs. LiPBE	LiPBE-med vs. HiRIT
**Cortical thickness**																			
FN total	1.467 ± 0.018	1.476 ± 0.020	0.7 ± 0.8 (-0.8, 2.3)	1.477 ± 0.019	1.499 ± 0.021	1.4 ± 0.8 (-0.3, 3.0)	1.455 ± 0.035	1.485 ± 0.029	2.3 ± 1.5 (-0.8, 5.3)	1.397 ± 0.027	1.452 ± 0.041 *	3.9 ± 1.6 (0.6, 7.1)	0.367	1.000	1.000	1.000	1.000	0.546	1.000
FN medial	2.423 ±0.034	2.478 ±0.038 *	2.4 ± 0.9 (0.6, 4.1)	2.462 ± 0.037	2.514 ± 0.041 *	2.0 ± 1.0 (0.1, 3.9)	2.429 ± 0.067	2.424 ± 0.076	0.1 ± 1.7 (-3.4, 3.6)	2.347 ± 0.071	2.437 ± 0.080 *	3.9 ± 1.8 (0.2, 7.5)	0.499	1.000	0.822	1.000	1.000	1.000	1.000
FN lateral	0.960 ± 0.015	0.944 ± 0.018	-1.3 ± 1.0 (-3.4, 0.7)	0.975 ± 0.016	0.986 ± 0.019	1.0 ± 1.1 (1.2, 3.2)	0.964 ±0.030	1.013 ± 0.035 *	4.8 ± 2.0 (0.8, 8.9)	0.915 ± 0.032	0.961 ±0.037 *	4.9 ± 2.2 (0.6, 9.1)	**0.012**	0.740	1.000	0.721	**0.050**	0.072	0.622
TH total	1.748 ± 0.016	1.765 ± 0.017 *	1.0 ± 0.5 (0.1, 2.0)	1.733 ± 0.018	1.737 ± 0.018	0.2 ± 0.5 (-0.7, 1.2)	1.727 ± 0.032	1.735 ± 0.033	0.5 ± 0.9 (-1.3, 2.3)	1.708 ± 0.034	1.717 ± 0.035	0.5 ± 1.0 (-1.4, 2.4)	0.693	1.000	1.000	1.000	1.000	1.000	1.000
**Cross-sectional**																			
CSA	0.759 ± 1.017	0.755 ± 0.017	-0.3 ± 0.8 (-2.0, 1.3)	0.759 ± 0.019	0.764 ± 0.018	0.6 ± 0.9 (-1.2 ± 2.4)	0.724 ± 0.034	0.735 ± 0.034	1.9 ± 1.6 (-1.4, 5.1)	0.732 ± 0.036	0.770 ± 0.036 *	6.1 ± 1.7 (2.7, 9.6)	**0.014**	1.000	0.462	**0.039**	1.000	**0.008**	1.000
CSMI	0.970 ± 0.030	0.971 ± 0.030	0.6 ± 1.0 (-1.4, 2.7)	0.990 ± 0.032	1.007 ± 0.032	1.7 ± 1.1 (-0.6, 3.9)	0.930 ± 0.059	0.944 ± 0.059	1.8 ± 2.1 (-2.3, 5.8)	0.867 ± 0.062	0.914 ± 0.062 *	5.8 ± 2.2 (1.5 ± 10.1)	0.291	1.000	1.000	0.586	1.000	0.219	1.000
Z	0.531 ± 0.014	0.523 ± 0.014	-1.3 ± 1.0 (-3.3, 0.7)	0.533 ± 0.015	0.539 ± 0.015	1.0 ± 1.1 (-1.2, 3.2)	0.500 ± 0.027	0.515 ± 0.027	3.2 ± 2.0 (-0.7 ± 7.2)	0.488 ± 0.028	0.522 ± 0.029 *	7.6 ± 2.1 (3.4, 11.8)	**0.002**	0.744	0.830	**0.047**	0.280	**0.002**	1.000

Abbreviations: CI, confidence interval; CSA, cross-sectional area; CSMI, cross-sectional moment of inertia; FN, femoral neck; HiRIT, high-intensity resistance and impact training; HiRIT-med, high-intensity resistance and impact training plus medications; ITT, intention-to-treat; LiPBE, low-intensity Pilates-based exercise; LiPBE-med, low-intensity Pilates-based exercise plus medications; TH, total hip; Z, section modulus
Data are mean ± SE
p values represent between-group comparison of % change from one-way ANCOVA, adjusted for age at baseline
* Within-group change from baseline $p \leq 0.05$ from RMANCOVA
